# Genome Sequence of *Bacillus anthracis* Strain Stendal, Isolated from an Anthrax Outbreak in Cattle in Germany

**DOI:** 10.1128/genomeA.00219-16

**Published:** 2016-04-07

**Authors:** Markus Antwerpen, Mandy Elschner, Wolfgang Gaede, Annette Schliephake, Gregor Grass, Herbert Tomaso

**Affiliations:** a Bundeswehr Institute of Microbiology, Munich, Germany; b Friedrich-Loeffler-Institut, Institute of Bacterial Infections and Zoonoses, Jena, Germany; c Department of Veterinary Medicine, State Institute for Consumer Protection of Saxony-Anhalt, Stendal, Germany

## Abstract

In July 2012, an anthrax outbreak occurred among cattle in northern Germany resulting in ten losses. Here, we report the draft genome sequence of *Bacillus anthracis* strain Stendal, isolated from one of the diseased cows.

## GENOME ANNOUNCEMENT

The zoonotic disease anthrax caused by *Bacillus anthracis* is a rare disease in Germany, with only three reported outbreaks between 2009 and the end of 2015 (1, 2). An outbreak early in July 2012 occurred among livestock in the county of Stendal (federal state of Saxony-Anhalt) resulting in livestock loss of 10 out of 55 cows (2). As a consequence, 50 people received postexposure prophylaxis with antibiotic therapy, and the affected pasture was decontaminated with formalin. This outbreak received considerable media attention because one of the diseased cows had died at the shores of the river Elbe and was swept away by the current.

Strain Stendal 1 was isolated from blood culture of one of the diseased cows, and the presence of both virulence plasmids pXO1 and pXO2 of *B. anthracis* was confirmed by real-time PCR assays (3, 4). Canonical single-nucleotide polymorphism (canSNP)–based genotyping (5) revealed that the outbreak strain belonged to the branch A.Br.001/002, which has previously been isolated in Germany and its neighboring countries. Indeed, it appears that this canSNP-genotype of *B. anthracis* is predominant in Germany, Belgium, the Netherlands, Denmark, and Norway (6).

Whole-genome shotgun (WGS) sequencing of *B. anthracis* Stendal was performed by Ion Torrent sequencing technology (Ion Torrent Systems Inc., USA). For the WGS library, 1,705,145 reads with a total of 460 Mb were generated. Bowtie-2 (7) was used for mapping to the Ames Ancestor chromosome, plasmids pXO1 and pXO2 (NC_007530.2, NC_007322.2, AE017335.3), and the sequence was amended by multilocus variable-number of tandem-repeat analysis data according to (8), where necessary. The G+C content was calculated using an in-house Python script.

The total length of the genome shotgun sequence of *B. anthracis* Stendal was 5,227,419 bp with a 63-fold coverage for the chromosome (137-fold for pXO1 and 103-fold for pXO2), and the mean G+C content was 35.5%. For initial annotation, the assembled contigs were submitted to the RAST annotation pipeline (9). The *B. anthracis* Stendal draft genome encodes 5,639 putative coding sequences. Annotation of the genome identified 11 copies of genes for the 16S rRNA, the 5S rRNA, and the 23S rRNA; 61 tRNA loci were identified. The *B. anthracis* Stendal genome represents a third major genotype, A.Br.001/002, of this bacterium from rare outbreaks in Germany in this millennium. The other two were strain UR-1, canSNP-group A.Br.008/011, from a diseased heroin consumer in Bavaria (10), and strain BF-1, canSNP-group B.Br.001/002, from a dead cow of the Bavarian Alps region (1).

### Nucleotide sequence accession numbers.

This whole-genome shotgun project has been deposited at DDBJ/EMBL/GenBank under the accession numbers CP014177 (pXO1), CP014178 (pXO2), and CP014179 (chromosome). The versions described in this paper are the first versions.
